# Simulation of a medication and methylation effects on triglycerides in the Genetic Analysis Workshop 20

**DOI:** 10.1186/s12919-018-0115-z

**Published:** 2018-09-17

**Authors:** Aldi T. Kraja, Ping An, Petra Lenzini, Shiou J. Lin, Christine Williams, James E. Hicks, E. Warwick Daw, Michael A. Province

**Affiliations:** Division of Statistical Genomics, Department of Genetics and Center for Genome Sciences and Systems Biology, Washington University School of Medicine, 4523 Clayton Ave, Saint Louis, MO 63110 USA

## Abstract

The GAW20 simulation data set is based upon the companion Genetics of Lipid Lowering Drugs and Diet Network (GOLDN) study fenofibrate clinical trial data set that forms the real data example for GAW20. The simulated data problem consists of 200 simulated replications of what might happen if we were to repeat the GOLDN clinical trial 200 independent times, for these exact same subjects, but using a new fictitious drug (called “genomethate”) that has a pharmaco-epigenetic effect on triglyceride response. For each replication, the pre-genomethate values at visits 1 and 2 are constant (ie, pedigree structures, age, sex, all phenotypes, covariates, genome-wide association study (GWAS) genotypes, and visit 2 methylation values), the same as the real GOLDN data across all 200 replications. Only the post-genomethate treatment data (ie, methylation and triglyceride levels for visits 3 and 4) change across the 200 replications. We postulate a growth curve pharmaco-epigenetic response model, in which each patient’s response to genomethate treatment is individualized, and is dependent upon their genotype as well as the methylation state for key genes.

## Background

The companion Genetics of Lipid Lowering Drugs and Diet Network (GOLDN) study fenofibrate clinical trial data set [1–3] was the foundation of our Genetic Analysis Workshop 20 (GAW20) simulation. The general simulation strategy was to first simulate visit 4 methylation array data for each subject (which measures the individual epigenetic responses to genomethate treatment), and then use this plus the genome-wide association study (GWAS) genotypes to produce the simulated triglycerides for visits 3 and 4 post-treatment values. The main simulated effect of genomethate is on the phenotype of the individual subject’s triglyceride (TG) values measured as slope in response to treatment (change in mg/dL per unit time of treatment).

## Methods, results and discussion

**Fig. 1:**
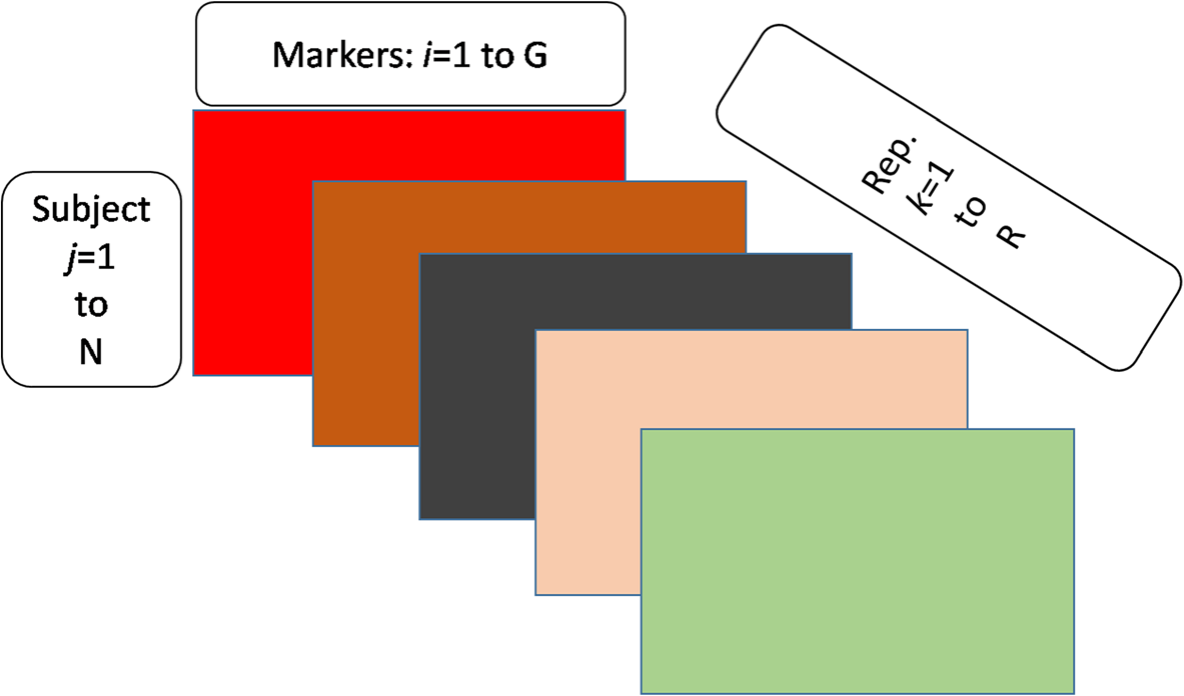
A 3D indexing order of the GAW20 simulation. The *j* index in the figure represents the subjects, the *i* index is noting the causal SNPs, where *i* = 1–5 also indexes the 5 main effects of the corresponding nearby CpG sites, while the sites 6–105 are 100 SNPs with background genetic effects. The *k* index indicates replications (*k* = 1, 2, …, *R* = 200)

Figure 1 illustrates the graphical design of the simulations.

The *j* index in the figure represents the subject (*j* = 1, 2, …, *N* = 717). The *i* index is noting the single-nucleotide polymorphisms (SNPs) chosen to be causal in the simulating model (*i* = 1, 2, …, G = 105), where *i* = 1, 2, 3, 4, 5 also indexes the 5 main effects of the corresponding nearby cytosine-phosphate-guanine (CpG) sites, while beyond main effects, the sites from 6 to 105 are 100 SNPs with background genetic effects. The *k* index indicates replications (*k* = 1, 2, …, *R* = 200).

The first 5 causal SNPs are “major” effects (summarized in Table 1), and the last 100 SNPs are polygenic background effects (Table 2). Note that only the first 5 CpG sites are relevant to the model, the polygenic background effects do *not* depend upon CpG states.

**Table 1:** Five major effect causal SNPs and corresponding nearby CpG markers affecting triglycerides at visits 3 and 4

Methylvar	chrom	cgposition	cggene	CpG	CpG	Cp	CpG	markname	chrom	rsposition	rsgene	role	hg^2^	diffpos
				mean	Sd	Gmean	Sd							
				V2	V2	V4	V4							
cg00000363	1	230560793		0.488	0.0589	0.492	0.3273	rs9661059	1	230556033			0.125	− 4760
cg10480950	6	5067127		0.578	0.0571	0.56	0.3247	rs736004	6	5067728	LYRM4	intron	0.075	601
cg18772399	8	89478349		0.575	0.0743	0.556	0.3265	rs1012116	8	89466383			0.100	−11966
cg00045910	10	23466070		0.474	0.0896	0.482	0.3295	rs10828412	10	23476515			0.025	10445
cg01242676	17	13413600	HS3ST3A1	0.456	0.0837	0.464	0.328	rs4399565	17	13407619	HS3ST3A1	intron	0.050	− 5981

*Abbreviations:* methylvar, CpG marker name; chrom, CpG marker’s chromosome; cgposition, CpG marker position in base pairs; cggene, CpG marker’s gene; CpGmeanV2, mean of methylation at visit 2; CpGSdV2, standard deviation of the same methylation marker at visit 2; CpGmeanV4, mean of methylation at visit 4; CpGSdV4, standard deviation of the same methylation marker at visit 4; markname, SNP name; chrom, SNP’s chromosome; rsposition, SNP’s position in base pairs; rsgene, SNP’s gene name; role, SNP’s role; hg^2^, simulated expected heritability for each causative SNP; diffpos, difference in base pair positions between corresponding SNP and CpG markers

**Table 2:** Background polygenic SNPs. All markers are simulated with the same heritability (hg^2^ = 0.001) affecting triglycerides at visits 3 and 4

markname	chrom	position	Gene	role	strand_affy	allele_affy	coded_all	noncoded_all	coded_af	P_HWE	Callrate	SNPID
rs12037545	1	14875400	KIAA1026	intron	–	A/G	G	A	0.51764	0.378	1	SNP_A_2245928
rs11102122	1	1.11E + 08			+	G/T	T	G	0.534672	0.5963	0.997625	SNP_A_8451677
rs2004659	1	1.44E + 08	NUDT17	intron	–	A/G	A	G	0.629111	0.2478	0.995249	SNP_A_2211220
rs3806218	1	1.45E + 08	BCL9	near-g	+	C/T	C	T	0.655718	0.1067	0.998812	SNP_A_8575283
rs2352866	1	1.46E + 08			+	C/T	T	C	0.980535	1	0.998812	SNP_A_8635861
rs4637157	2	19443			+	C/T	T	C	0.906326	1	1	SNP_A_8500963
rs11903036	2	584324			–	G/T	G	T	0.53163	0.7181	1	SNP_A_8639506
rs4549126	2	714368			–	C/T	T	C	0.614964	0.448	1	SNP_A_8430739
rs6758300	2	75483393			–	A/G	G	A	0.568735	0.8567	1	SNP_A_2284162
rs4667937	2	1.67E + 08			–	A/T	A	T	0.739659	1	1	SNP_A_1851057
rs6785370	3	3933764			+	A/G	A	G	0.874696	0.4383	0.996437	SNP_A_4202617
rs7628979	3	4321347	SETMAR	intron	+	G/T	T	G	0.529197	0.07716	1	SNP_A_4290525
rs711664	3	4437544	SUMF1	intron	+	C/T	C	T	0.706204	0.6558	1	SNP_A_2098242
rs35489229	3	5088187			–	A/G	A	G	0.892336	1	1	SNP_A_2299978
rs1524557	3	81974721			–	A/G	A	G	0.639903	0.4311	1	SNP_A_2123904
rs1466475	4	80175727			–	C/T	T	C	0.81691	0.01551	1	SNP_A_2063157
rs2615479	4	88797334	DMP1	intron	–	C/T	C	T	0.71837	1	1	SNP_A_1862590
rs6849123	4	91684534	MGC48628	intron	–	A/G	G	A	0.894769	1	1	SNP_A_8414444
rs4267808	4	1.1E + 08	COL25A1	intron	–	G/T	T	G	0.871655	1	1	SNP_A_2063127
rs9992755	4	1.11E + 08	EGF	intron	+	A/G	A	G	0.686131	0.5216	1	SNP_A_1873873
rs11951861	5	84715767			–	A/G	A	G	0.827251	0.5626	1	SNP_A_8538997
rs1428900	5	84909967			–	C/T	C	T	0.731144	0.2558	1	SNP_A_2241509
rs17207011	5	85486010			+	A/C	C	A	0.886861	1	1	SNP_A_8651237
rs372106	5	1.23E + 08			+	A/G	G	A	0.619221	0.8545	1	SNP_A_1850833
rs7730187	5	1.68E + 08	SLIT3	intron	+	C/T	C	T	0.518248	0.1095	1	SNP_A_4282886
rs1482570	6	72738670	RIMS1	intron	–	C/G	C	G	0.867397	0.4147	1	SNP_A_2147869
rs1281958	6	1.53E + 08			–	G/T	G	T	0.587591	0.8568	1	SNP_A_4222639
rs9479769	6	1.55E + 08	OPRM1	intron	–	A/C	A	C	0.546837	0.5862	1	SNP_A_2252195
rs9322560	6	1.56E + 08			+	A/G	A	G	0.857664	0.7365	1	SNP_A_2204739
rs9457675	6	1.6E + 08			–	G/T	G	T	0.641728	0.8483	1	SNP_A_2109851
rs4721428	7	2137132	MAD1L1	intron	+	A/G	A	G	0.678832	0.03313	1	SNP_A_8644552
rs6461984	7	3314009	SDK1	intron	+	A/G	A	G	0.703771	1	0.997625	SNP_A_8500870
rs17186478	7	5779274	RNF216	intron	–	A/C	C	A	0.603406	0.8521	1	SNP_A_2276119
rs2110333	7	8151614	ICA1	intron	+	C/T	T	C	0.708029	0.09075	1	SNP_A_8699267
rs1352090	7	46160368			–	C/G	G	C	0.615572	0.364	1	SNP_A_8478994
rs4733163	8	33653826			–	A/G	G	A	0.625304	0.09864	1	SNP_A_8357393
rs2981182	8	40010613			–	A/C	C	A	0.53528	0.03005	1	SNP_A_8501882
rs2923408	8	42570683			+	A/G	A	G	0.542579	0.7232	1	SNP_A_2264082
rs16921991	8	58386566			+	C/T	T	C	0.796837	0.2484	1	SNP_A_4212967
rs10955119	8	98468181			–	C/G	G	C	0.58455	0.01847	1	SNP_A_1873431
rs7036143	9	90615114			–	C/T	T	C	0.827859	1	1	SNP_A_8332992
rs2196921	9	91748045			–	C/T	C	T	0.81691	0.4025	1	SNP_A_8714258
rs12238738	9	95433628	PHF2	intron	–	C/T	T	C	0.622263	0.02467	1	SNP_A_2103415
rs10984103	9	99679096			–	G/T	G	T	0.652068	1	1	SNP_A_8701456
rs1989773	9	1.17E + 08			+	A/G	G	A	0.990876	1	1	SNP_A_8526939
rs10887185	10	85670555			+	C/G	C	G	0.914234	0.09797	1	SNP_A_1799218
rs481179	10	1.08E + 08			+	C/T	T	C	0.576642	0.7135	1	SNP_A_8706770
rs17586536	10	1.2E + 08	C10orf46	intron	+	C/T	T	C	0.639903	1	1	SNP_A_8582485
rs10788015	10	1.22E + 08			–	C/T	T	C	0.569343	0.7223	1	SNP_A_8527983
rs4339955	10	1.22E + 08			+	C/T	T	C	0.78528	0.8055	1	SNP_A_8334121
rs11030861	11	29853551			–	A/G	G	A	0.937956	1	1	SNP_A_1788514
rs7947279	11	82018398			+	C/T	T	C	0.893552	0.1277	1	SNP_A_8345915
rs10895219	11	1.01E + 08	ANGPTL5	intron	–	C/T	T	C	0.965937	1	1	SNP_A_8703545
rs9888281	11	1.26E + 08	KIRREL3	intron	–	G/T	T	G	0.799878	0.5658	1	SNP_A_2206833
rs10790956	11	1.28E + 08	ETS1	intron	–	C/T	C	T	0.565085	1	1	SNP_A_2253706
rs7138234	12	21569984	C12orf39	near-g	+	C/T	T	C	0.994526	1	1	SNP_A_4277693
rs12426560	12	41977227			–	C/T	C	T	0.814477	0.2236	1	SNP_A_8404603
rs11183911	12	46055518			+	A/G	A	G	0.818735	0.7645	1	SNP_A_1956756
rs11113259	12	1.06E + 08			+	C/T	C	T	0.999392	1	0.998812	SNP_A_2030476
rs10219441	12	1.15E + 08			–	C/T	T	C	0.692214	0.5258	1	SNP_A_4223101
rs4427687	13	73781335			–	G/T	G	T	0.757299	0.1453	0.998812	SNP_A_2003390
rs9318328	13	74726372			+	A/T	T	A	0.541971	0.8591	0.998812	SNP_A_1854478
rs9573791	13	75607873			–	A/G	A	G	0.902068	1	1	SNP_A_2160026
rs2329072	13	77858815			+	C/T	T	C	0.594282	0.6994	1	SNP_A_2162287
rs2633019	13	82465113			–	A/G	A	G	0.530414	1	1	SNP_A_2024048
rs12897163	14	59385513	RTN1	intron	+	A/C	A	C	0.751217	0.3606	1	SNP_A_2233795
rs2121063	14	75798908			+	C/G	G	C	0.893552	0.2988	1	SNP_A_4298064
rs1676295	14	76103995			–	C/G	C	G	0.789538	1	1	SNP_A_2003847
rs1430569	14	86878849			–	C/T	C	T	0.53528	0.374	1	SNP_A_2185553
rs6575695	14	98363450			–	C/T	C	T	0.973236	1	1	SNP_A_2133926
rs1390876	15	45433081			–	C/T	C	T	0.595499	0.06189	1	SNP_A_2277486
rs13313462	15	45534066			–	C/T	T	C	0.620438	0.8445	1	SNP_A_8421063
rs7180426	15	60558330			–	C/T	C	T	0.859489	1	1	SNP_A_8713038
rs17477813	15	76147746	TBC1D2B	intron	–	C/T	T	C	0.692214	0.831	1	SNP_A_8395279
rs2072986	16	1631107	CRAMP1L	intron	–	A/G	A	G	0.847324	1	1	SNP_A_8348939
rs1077836	16	10248642			–	G/T	T	G	0.66545	0.7045	1	SNP_A_2287848
rs8052975	16	10856764			+	C/T	C	T	0.711679	1	1	SNP_A_8486961
rs6497651	16	23040046	USP31	intron	+	C/T	C	T	0.975669	1	1	SNP_A_8525616
rs27817	16	48013792			+	A/G	G	A	0.97202	1	0.997625	SNP_A_8365337
rs9897174	17	49611224			+	G/T	G	T	0.948905	1	1	SNP_A_2309201
rs345168	17	55565566			–	A/G	G	A	0.959854	1	1	SNP_A_1796579
rs9908999	17	56215981	BCAS3	intron	+	A/G	A	G	0.909367	0.5936	1	SNP_A_1848643
rs1112364	17	57597131			+	C/T	C	T	0.905718	0.4362	1	SNP_A_8550334
rs12936559	17	57680004			+	A/G	G	A	0.935523	1	1	SNP_A_8410067
rs1318841	18	17138521			–	C/T	C	T	0.967153	1	1	SNP_A_2087816
rs17202807	18	19434594	ANKRD29	utr-3	+	G/T	T	G	0.955596	1	0.998812	SNP_A_2132404
rs339869	18	20461587			+	A/G	A	G	0.55292	0.4779	1	SNP_A_8437981
rs11083025	18	49698325			–	A/G	G	A	0.930657	1	1	SNP_A_1888265
rs4325666	18	65460176	DOK6	intron	–	C/T	C	T	0.933698	1	1	SNP_A_1924329
rs8111862	19	12420713			–	C/T	C	T	0.51399	0.5942	1	SNP_A_2179593
rs2453888	19	22423985			–	C/G	G	C	0.849757	0.03764	1	SNP_A_8549134
rs16999009	19	22701498			+	A/G	G	A	0.877129	0.2534	1	SNP_A_2094893
rs7252281	19	35965262			+	G/T	T	G	0.65204	0.1049	0.98337	SNP_A_1867428
rs7254832	19	43637691	RYR1	intron	+	C/T	C	T	0.871046	0.7148	1	SNP_A_1791707
rs1974821	19	56609547	LOC10012	coding	–	C/T	C	T	0.852798	1	1	SNP_A_8587419
rs6056690	20	9475353	PAK7	intron	–	C/T	T	C	0.850365	0.1277	1	SNP_A_2250060
rs1415774	20	33229277	PROCR	near-g	–	C/T	C	T	0.566302	0.4777	1	SNP_A_2130084
rs6093657	20	40549705	PTPRT	intron	+	A/G	A	G	0.877737	1	1	SNP_A_8463206
rs7260668	20	42919440			–	A/G	A	G	0.694039	0.8358	1	SNP_A_1875543
rs13042657	20	44356316			–	C/T	C	T	0.784063	0.7955	1	SNP_A_8623899

*Abbreviations:* markname, SNP name; chrom, SNP’s chromosome; position, SNP’s position in base pairs; Gene, SNP’s gene name; role, SNP’s role; strand_affy, +/− strand of the SNP; allele_affy, the SNP’s Affymetrix array alleles; coded_all, coded allele; noncoded_all, noncoded allele; coded_af, coded allele frequency; P_HWE, *p*-value for testing Hardy Weinberg Equilibrium; Callrate, call rate for the SNP; SNPID, Affymetrix array SNP ID

We first defined a series of subjects’ triglyceride values from the original (real) Genetics of Lipid Lowering Drugs and Diet Network (GOLDN) data [1], which was used to generate the simulations. Because triglycerides were approximately log-normally distributed, we worked with log-transformed triglyceride values in all calculations, only transforming back to the measured triglyceride scale at the end of the simulations. In particular, for the *j*th subject, the average log triglycerides pre-treatment (average of visits 1 and 2, which are 1 day apart) and post-treatment (average of visits 3 and 4, which are also 1 day apart) in the original (real) GOLDN data are:

$$ \boldsymbol{O}\_\boldsymbol{preRx}\_{\boldsymbol{TG}}_{\boldsymbol{j}}=\boldsymbol{mean}\left(\mathbf{\log}\left(\boldsymbol{TG}{\mathbf{1}}_{\boldsymbol{j}}\right),\mathbf{\log}\left(\boldsymbol{TG}{\mathbf{2}}_{\boldsymbol{j}}\right)\right) $$

$$ \boldsymbol{O}\_\boldsymbol{postRx}\_{\boldsymbol{TG}}_{\boldsymbol{j}}=\boldsymbol{mean}\left(\mathbf{\log}\left(\boldsymbol{TG}{\mathbf{3}}_{\boldsymbol{j}}\right),\mathbf{\log}\left(\boldsymbol{TG}{\mathbf{4}}_{\boldsymbol{j}}\right)\right) $$

where ***O*** –stands for “Observed / Original”, ***preRx*** stands for “pre-medication treatment,” ***postRx*** stands for “after medication treatment,” and ***TG*** labels “triglycerides” which were log transformed to ensure a normal distribution of the trait. The ***TG*** of person *j* is measured in visits 1, 2, 3 and 4 and averaged as above for each individual as ***preRx*** and ***postRx***. The corresponding change in log triglycerides pre-treatment to post-treatment for subject *j* is given by:

$$ \boldsymbol{O}\_\boldsymbol{delta}\_{\boldsymbol{TG}}_{\boldsymbol{j}}=\left[\boldsymbol{O}\_\boldsymbol{postRx}\_{\boldsymbol{TG}}_{\boldsymbol{j}}-\boldsymbol{O}\_\boldsymbol{preRx}\_{\boldsymbol{TG}}_{\boldsymbol{j}}\right] $$

where ***delta*** is the “change”. The individual time on treatment (less than 30 days) for each subject (in days), is given by the following formula:

$$ \boldsymbol{O}\_{\boldsymbol{daysRx}}_{\boldsymbol{j}}=\boldsymbol{mean}\left(\boldsymbol{draw}\_\boldsymbol{date}\_\boldsymbol{v}{\mathbf{3}}_{\boldsymbol{j}},\boldsymbol{draw}\_\boldsymbol{date}\_\boldsymbol{v}{\mathbf{4}}_{\boldsymbol{j}}\right)-\boldsymbol{draw}\_\boldsymbol{date}\_\boldsymbol{v}{\mathbf{2}}_{\boldsymbol{j}} $$

where ***daysRx*** is “days after medication treatment,” ***draw***_***date*** is “blood draw date” at a particular ***v*- “**visit.” Thus, the observed slope (change in log triglycerides over the treatment period) is:

$$ \boldsymbol{O}\_\boldsymbol{slope}\_{\boldsymbol{TG}}_{\boldsymbol{j}}=\boldsymbol{O}\_\boldsymbol{delta}\_{\boldsymbol{TG}}_{\boldsymbol{j}}/\boldsymbol{O}\_{\boldsymbol{daysRx}}_{\boldsymbol{j}} $$

If ***mean***_***O***_***PreRx***_***TG*** and ***sd***_***O***_***preRx***_***TG*** are the mean and standard deviations, respectively, of all the ***O***_***preRx***_***TG***_***j***_ across the *j* = 1, …, N individuals, then the standardized original ***preRx*** of ***TG***_***j***_ are given by:

$$ \boldsymbol{O}\_{\boldsymbol{preZ}}_{\boldsymbol{j}}=\left(\boldsymbol{O}\_\boldsymbol{preRx}\_{\boldsymbol{TG}}_{\boldsymbol{j}}-\boldsymbol{mean}\_\boldsymbol{O}\_\boldsymbol{PreRx}\_\boldsymbol{TG}\right)/\boldsymbol{sd}\_\boldsymbol{O}\_\boldsymbol{preRx}\_\boldsymbol{TG} $$

where ***O***_***preZ*** -is a standardized normally distributed variable with *N(0,1)*.

Tables 1 and 2 summarize the epigenetic model in our simulation. We chose 5 “major gene” causal variants (ranging from modest to small effect sizes corresponding to expected “heritabilities” of 0.125, 0.10, 0.075, 0.05, and 0.025), which, in the absence of any epigenetic effects, should govern individual genomethate treatment response along with 100 polygene variants (each of tiny effect size corresponding to “heritabilities” of 0.001 each). These were chosen randomly from chromosomes 1–20 of the GWAS Affymetrix Genome-wide Human SNP Array 6.0, which had 718,544 autosomal SNPs.

For the epigenetic component, we choose 5 CpG sites on the Illumina Infinium HumanMethylation450 BeadChip array (which had 463,995 CpG sites) that are physically closest to the 5 “major gene” causal SNPs, while the methylation sites near the 100 polygenes have no effect. The genomethate response model is based upon the idea that these CpG sites need to be sufficiently unmethylated for the corresponding causal SNPs to express their influence on each individual’s phenotype. If the nearby CpG site is totally methylated (=1), then the corresponding causal SNP actually has *no* effect on the phenotype. If the CpG site is totally unmethylated (=0), then the corresponding causal SNP carries its full effect size impact on the phenotype. If the CpG site is partially methylated (between 0 and 1), then the effect size of the causal SNP is proportionally attenuated.

Specifically, for the *k*th simulation, we first generated the simulated visit 4 methylation array results for all subjects, based upon their corresponding visit 2 and/ or visit 4 methylation values. For each subject *j* = 1, …, 717, and each CpG methylation site *i* = 1, 2, 3, 4, 5 (corresponding to 5 major effect CpGs)

$$ \boldsymbol{sim}\_\boldsymbol{meth}\_\boldsymbol{v}{\mathbf{4}}_{\boldsymbol{ji}\boldsymbol{k}}=\boldsymbol{real}\_\boldsymbol{meth}\_\boldsymbol{v}{\mathbf{2}}_{\boldsymbol{ji}}+{\boldsymbol{sd}}_{\boldsymbol{i}}\ast \boldsymbol{Z}{\mathbf{1}}_{\boldsymbol{ji}\boldsymbol{k}} $$

where ***sim***_***meth*** stands for “simulated methylation” at visit 4, ***real***_***meth*** is the *j*th subject’s “real methylation” array data at visit 2 for the *i*th CpG site, ***sd***_***i***_ = 0.4 represents the standard deviation of individual subject methylation responses to treatment, and ***Z*1*jik*** *~ N(0, 1)* is a pseudo-random standard normal variable drawn independently for each *jik*.

For the remaining, non-causal CpG sites, if the subject *j* had real visit 4 methylation array data then

$$ \boldsymbol{sim}\_\boldsymbol{meth}\_\boldsymbol{v}{\mathbf{4}}_{\boldsymbol{ji}\boldsymbol{k}}=\boldsymbol{real}\_\boldsymbol{meth}\_\boldsymbol{v}{\mathbf{4}}_{\boldsymbol{ji}}+{\boldsymbol{sd}}_{\boldsymbol{i}}\ast \boldsymbol{Z}{\mathbf{1}}_{\boldsymbol{ji}\boldsymbol{k}} $$

Otherwise, if the subject *j* only had visit 2 methylation array data, then

$$ \boldsymbol{sim}\_\boldsymbol{meth}\_\boldsymbol{v}{\mathbf{4}}_{\boldsymbol{ji}\boldsymbol{k}}=\boldsymbol{real}\_\boldsymbol{meth}\_\boldsymbol{v}{\mathbf{2}}_{\boldsymbol{ji}}+{\boldsymbol{sd}}_{\boldsymbol{i}}\ast \boldsymbol{Z}{\mathbf{1}}_{\boldsymbol{ji}\boldsymbol{k}} $$

where ***real***_***meth***_***v*2**_***ji***_ and ***real***_***meth***_***v*4**_***ji***_ are the real visit 2 and visit 4 methylation array data, respectively, for subject *j* and CpG site *i*, ***sd***_***i***_ represents the standard deviation of individual subject methylation responses to treatment for the *i*th CpG site, and again, ***Z*1*jik*** *~ N(0,1)* is a pseudo-random variable drawn independently for each *jik*.

We selected five random non-causal (red-herrings) CpG sites also (shown in Table 3). We set for them the ***sd***_***i***_ = 0.4, to be similar to the simulated causal CpG sites. For the remaining non-causal CpG sites, we set the corresponding ***sd***_***i***_ = 0.03, which is closer to that seen in the real visit 4 methylation data CpG sites, essentially at the measurement error level.

**Table 3:** Five non-causal (red-herrings) CpG markers chosen to have N(0,0.4) random variability, imitating the distribution of the 5 real causative CpG markers

Methylvar	chrom	cgposition	cggene	CpGdata Partition	rsid	rsposition	rsRole	rsGene	strand_affy	allele_affy	coded_allele	noncoded_all	coded_all_freq	p_HWE	callrate	snpid
cg00703276	3	1.3E + 08	NA	3	rs2953763	131243312		NA	–	A/G	G	A	0.987211	1	0.99881	SNP_A_8675856
cg01971676	7	4.3E + 07	HECW1	8	rs6960763	43150741	intron	HECW1	+	C/T	C	T	0.550983	0.017	0.98931	SNP_A_2264336
cg11736230	14	1E + 08	PPP1R13B	43	rs2494731	104308725	intron	AKT1	+	C/G	G	C	0.677045	0.6804	0.99406	SNP_A_2232252
cg00001261	16	3463964	NA	1	rs4786421	3462304	intron	FLJ14154	+	A/G	G	A	0.690389	0.0168	1	SNP_A_4291807
cg12598270	18	3.3E + 07	ZNF396	46	rs323312	32996624	intron	KIAA1328	–	A/G	G	A	0.858364	0.154	0.99525	SNP_A_4288135

*Abbreviations:* Methylvar, CpG marker name; chrom, CpG marker’s chromosome; cgposition, CpG marker position in base pairs; cggene, CpG marker’s gene; CpGdata Partition, a number that refers to a simulated data partition distributed; rsid, SNP name; rsposition, SNP’s position in base pairs; rsRole, SNP’s role; rsGene, SNP’s gene name; strand_affy, +/− strand on which the SNP is located; allele_affy – the SNP’s Affymetrix array alleles; coded_allele – coded allele; noncoded_all – noncoded allele; coded_all_freq – coded allele frequency; p_HWE – p-value for testing Hardy Weinberg Equilibrium; callrate – call rate for the SNP; snpid – Affymetrix array SNP ID

In all cases, *all* simulated visit 4 methylation values were then truncated to be strictly in the [0,1] interval, that is,

if (***sim***_***meth***_***v*4**_***jik***_ ***>*** **1**) then ***sim***_***meth***_***v*4**_***jik***_ ***=*** **1**

if (***sim***_***meth***_***v*4**_***jik***_ ***<*** **0**) then ***sim***_***meth***_***v*4**_***jik***_ ***=*** **0**

for all subjects *j*, CpG sites *i*, and simulation replications *k*.

Note that the model is such that, on average, the genomethate treatment has no effect on the amount of methylation increase/decrease from visit 2 to visit 4, however, there is variability across subjects. To reiterate, the variability is quite high (***sd***_***i***_ = 0.4) for the five CpG regions controlling the expression of the major causal variants and 5 other non-causal CpG (red-herrings) sites. The variability is low (***sd***_***i***_ = 0.03) for all other CpGs, at the level of measurement error.

Using these simulated visit 4 methylation data, we then generated the simulated slope change in triglyceride response for each individual *j* in each replication *k* as follows:

1 $$ {\boldsymbol{slope}}_{\boldsymbol{jk}}={\sum}_{\boldsymbol{i}=\mathbf{1}}^{\mathbf{5}}\left(\mathbf{1}-\boldsymbol{sim}\_\boldsymbol{meth}\_\boldsymbol{v}{\mathbf{4}}_{\boldsymbol{ji}\boldsymbol{k}}\right)\ast \boldsymbol{sqrt}\left(\boldsymbol{hg}{\mathbf{2}}_{\boldsymbol{i}}\right)\ast {\boldsymbol{SSNP}}_{\boldsymbol{ji}}+{\sum}_{\boldsymbol{i}=\mathbf{6}}^{\mathbf{105}}\boldsymbol{sqrt}\left(\boldsymbol{hg}{\mathbf{2}}_{\boldsymbol{i}}\right)\ast {\boldsymbol{SSNP}}_{\boldsymbol{ji}}+{\boldsymbol{zenv}}_{\boldsymbol{jk}}\ast \boldsymbol{sqrt}\left(\mathbf{1}-\sum \limits_{\boldsymbol{i}=\mathbf{1}}^{\mathbf{105}}\boldsymbol{hg}{\mathbf{2}}_{\boldsymbol{i}}\right) $$

In the above formula, ***zenv***_***jk***_ is an independently drawn pseudo-random normal deviate distributed *N(0,1)* for each subject *j* and each replication *k,* and it represents unexplained residual variation in the phenotype. ***SSNP***_***ji***_ is the standardized *i*th SNP additive genotype-dosage (i.e., coded such that mean = 0 and ***sd***_***i***_ = 1 in the sample), and the *i* = 1, 2, …, 105 regression coefficients in this linear model are given in terms of constants ***sqrt***(***hg*2**_*i*_*)*, in Tables 1 and 3. Note that if the five causal CpG sites were completely unmethylated for all subjects (i.e., no epigenetic effects), then (*1 –* ***sim***_***meth***_***v*4**_***j****ik*_) would be = 1 for all *j* = 1,…, N and *i* = 1,…, 5, and *k* = 1,…, 200, so that the regression coefficients would be interpreted as the square root of the locus specific heritability of the associated SNPs. Conversely, when the causal CpG site is totally methylated for that subject, (1 *-* ***sim***_***meth***_***v*4**_***j****ik*_) = 0, so that the corresponding major effect SNP_i_ will not express its effect on the phenotype. Similarly, if the CpG site is partially methylated (between 0 and 1), the effect size of the causal SNP is proportionally attenuated.

To carry forward these simulated relationships in eq. (1), we must address the fact that the observed slope responses for each subject are correlated to their baseline values of triglyceride (i.e., lower baseline values should produce less dramatic declines with treatment, whereas higher baseline values can experience greater slope change with treatment). In the real GOLDN data, the correlation between slope change in response to fenofibrate treatment and baseline log triglycerides is − 0.41881, and we used this constant value in our genomethate simulation to introduce a correlation between slope change and baseline values:

$$ {\boldsymbol{corrz}}_{\boldsymbol{j}\boldsymbol{k}}=\left(-\mathbf{0.41881}\right)\ast \boldsymbol{O}\_{\boldsymbol{preZ}}_{\boldsymbol{j}}+\boldsymbol{sqrt}\left(\mathbf{1}-{\left(\mathbf{0.41881}\right)}^{\mathbf{2}}\right)\ast {\boldsymbol{slope}}_{\boldsymbol{j}\boldsymbol{k}} $$

Because the simulated individual slopes are generated on the standardized scale, we needed to rescale to that of the original scale of triglyceride changes per day of treatment, by working backwards. The mean and standard deviation of ***O***_***slope***_***TG***_***j***_ over all subjects *j*, are denoted by ***mean***_***O***_***slope***_***TG*** and ***sd***_***O***_***slope***_***TG***, respectively. We used the above observed mean and standard deviation of slopes seen in the original GOLDN data, to rescale as follows:

$$ \boldsymbol{sim}\_{\boldsymbol{slope}}_{\boldsymbol{jk}}={\boldsymbol{corrz}}_{\boldsymbol{jk}}\ast \boldsymbol{sd}\_\boldsymbol{O}\_\boldsymbol{slope}\_\boldsymbol{TG}+\boldsymbol{mean}\_\boldsymbol{O}\_\boldsymbol{slope}\_\boldsymbol{TG} $$

Then the expected response to genomethate treatment of the *j*th subject, after ***O***_***DaysRx***_***j***_ original days of treatment, is given by:

$$ \boldsymbol{sim}\_\boldsymbol{postRx}\_{\boldsymbol{TG}}_{\boldsymbol{j}\boldsymbol{k}}=\left(\boldsymbol{sim}\_{\boldsymbol{slope}}_{\boldsymbol{j}\boldsymbol{k}}\ast \boldsymbol{O}\_{\boldsymbol{DaysRx}}_{\boldsymbol{j}}\right)+\boldsymbol{O}\_\boldsymbol{preRx}\_{\boldsymbol{TG}}_{\boldsymbol{j}} $$

Finally, we used the simulated individual responses to produce the simulated values of triglyceride at visits 3 and 4, based upon the variability we see between those visits in the real GOLDN fenofibrate data:

2 $$ \boldsymbol{sim}\_\boldsymbol{TG}{\mathbf{3}}_{\boldsymbol{j}\boldsymbol{k}}=\boldsymbol{\exp}\left[\boldsymbol{sim}\_\boldsymbol{postRx}\_{\boldsymbol{TG}}_{\boldsymbol{j}\boldsymbol{k}}+\left(\mathbf{\log}\Big(\boldsymbol{TG}{\mathbf{3}}_{\boldsymbol{j}}\right)-\boldsymbol{O}\_\boldsymbol{postRx}\_{\boldsymbol{TG}}_{\boldsymbol{j}}\Big)\right] $$

3 $$ \boldsymbol{sim}\_\boldsymbol{TG}{\mathbf{4}}_{\boldsymbol{j}\boldsymbol{k}}=\boldsymbol{\exp}\left[\boldsymbol{sim}\_\boldsymbol{postRx}\_{\boldsymbol{TG}}_{\boldsymbol{j}\boldsymbol{k}}+\left(\mathbf{\log}\Big(\boldsymbol{TG}{\mathbf{4}}_{\boldsymbol{j}}\right)-\boldsymbol{O}\_\boldsymbol{postRx}\_{\boldsymbol{TG}}_{\boldsymbol{j}}\Big)\right] $$

If only 1 replicate of the GAW20 simulated data was to be analyzed, we recommend the 84th replication, which was provided in a separate directory, as a “representative” of the 200 replicated simulations. Chromosomes 21 and 22 datasets were not used in the simulation, so an analyst can use the corresponding data for building a *NULL* hypothesis. The simulated GAW20 data are accompanied by README and Data Dictionary files.
